# Comment on “comparative analysis of triple-negative breast cancer transcriptomics of Kenyan, African American and Caucasian women” by Saleh et al.

**DOI:** 10.1016/j.tranon.2021.101164

**Published:** 2021-06-23

**Authors:** James P. Brody

**Affiliations:** Department of Biomedical Engineering, University of California, Irvine, Irvine, CA, United States

## Abstract

• Alterations to the somatic genome lead to a tumor.
• Different germ line genomes influence the formation of the tumor and its response to therapeutics.
• Germ line genetics can play a role in breast cancer.
• Race is an imperfect measure of germ line genetics.
• Black women have a higher incidence of triple negative breast cancer.

Cancer is a genetic disease. A single cell, which starts with the same germ line genome as the rest of the cells in the body, acquires genetic alterations and passes them onto its progeny. These somatic alterations, localized to the progeny of the initial cell, eventually form a tumor. To better understand cancers, we need to understand both why these alterations to the somatic genome lead to a tumor and how different germ line genomes influence the formation of the tumor and its response to therapeutics. In a recent paper, Saleh et al. [1] describe a natural experiment that provides a glimpse into how different germ line genomes respond to similar somatic changes in breast cancers. They compare molecular characteristics of triple negative breast tumors found in three categories of women: white and Black women from the US, and Bantu women from Kenya.

Somatic genome alterations in breast tumors affect the response of the tumor to therapies. The three most tracked somatic changes are alterations to the genes encoding the proteins known as ER (estrogen receptor), PR (progesterone receptor), and HER2 (a specific type of receptor tyrosine kinase). For instance, if a breast tumor contains the estrogen receptor (known as ER+ breast cancer), one common treatment is to use an anti-estrogen therapy, which selectively blocks the growth of these cancer cells and rapidly shrinks the tumor. Tumors that do not express the estrogen receptor do not react to anti-estrogen therapy. Breast tumors that lack all three of these receptors, known as triple negative breast cancer, are usually treated with non-specific chemotherapy.

Germ line genetics also can play a role in breast cancer. Inherited mutations in two different genes (BRCA1 and BRCA2) lead to increased risk of breast cancer. Most women with the BRCA1 mutations who develop breast cancer have triple negative breast cancer, while those with BRCA2 mutations end up with ER+ and/or PR+ forms of breast cancer [2]. These two inherited mutations account for less than 10% of all breast cancers in the United States. Single mutations have been exhaustively studied in breast cancer, but combinations of mutations have not. Many combinations of mutations, not yet known, contribute to breast cancer.

One imperfect measure of germ line genetics is race. Race and genetics are related in a complex way. One way to simplify this relationship is to consider where a person's ancestors lived a millennium ago, before the most recent migrations took place.

Humans first evolved in Africa and stayed there for millions of years. A simple, but useful, version of human history is this: The first archaic humans emerged in Africa 2 to 3 million years ago. About 50 to 100 thousand years ago, a few small groups left Africa and their descendants populated the rest of the world. The migration out of Africa was a bottleneck and has constrained the genetics of people outside of Africa. People inside of Africa have no such constraint. Over 90% of the period from the first humans to modern *Homo sapiens* occurred solely in Africa. Genetic surveys of different populations indicate that larger differences exist between different Africans than between Eurasians and some Africans [3].

A natural question is how germ-line genetics effects the molecular behavior of cells in a tumor. Race can be a proxy for germ line genetics to better understand this question. One approach is to compare breast cancer rates for white vs Black people in the United States. Because socioeconomic factors and race are intertwined in the United States, it is difficult to draw absolute conclusions about cancer rates in white people or Black people from this data. However, the socioeconomic effects can be partially factored out by comparing changes in cancer rates.

Fig. 1 shows the age-specific incidence of breast cancer as measured by the SEER-21 cancer registries in the US between 2000 and 2016. The figure shows that breast cancer is more common in Black women, as compared to white women, for women less than 40 years old. However, for women greater than 45 years old breast cancer is more common in white women than Black women. This effect is known as the Black-white crossover [4,5].

**Fig. 1:**
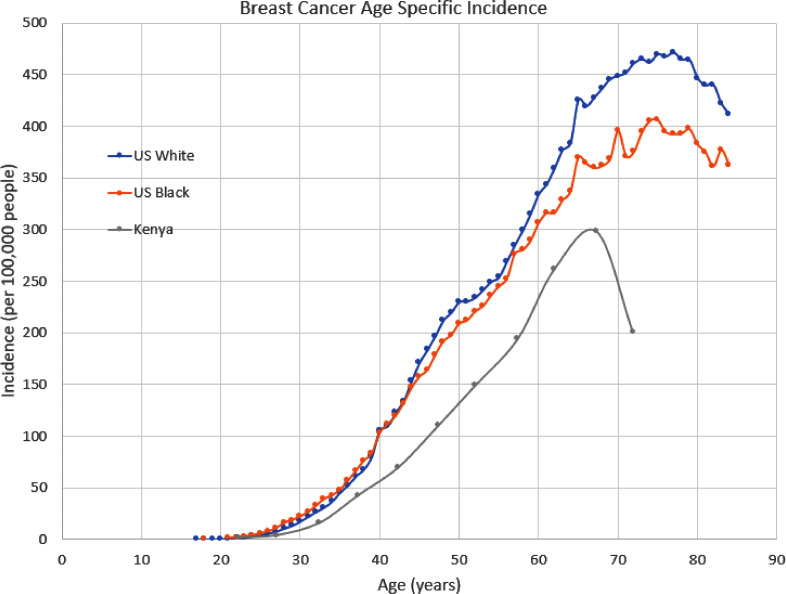
The age specific incidence for breast cancer measured in three distinct groups of women. The US data is reported by the SEER 21 cancer registries over the timespan from 2000 to 2016. The Kenya data is from the African Cancer Registry Network (2003 to 2014).

For comparison, Fig. 1 also includes the age specific incidence of breast cancer in Nairobi, Kenya [6]. The measured incidence in Kenya is lower at all ages for breast cancer, but this difference is not necessarily due to germ line genetics. People in Kenya are exposed to different environmental factors, have very different diets, and undergo screening at very different rates. For instance, early diagnosis shifts this curve shown in Fig. 1 to the left. If breast cancers are diagnosed two years later in Kenya than in the US, these curves would be much more similar. Similarly, environmental and dietary differences between Kenya and the US could also account for some of the differences in rates.

Fig. 2 shows similar age specific incidence data for triple negative breast cancers. Black women in the US have a significantly higher incidence of triple negative breast cancer at all ages. (Similar data on triple negative breast cancers in Kenya is not available.) The reason for this difference is not known but could be related to germ line genetics. While these population level statistics are informative, molecular level characterization would add significantly to our understanding of breast cancers..

**Fig. 2:**
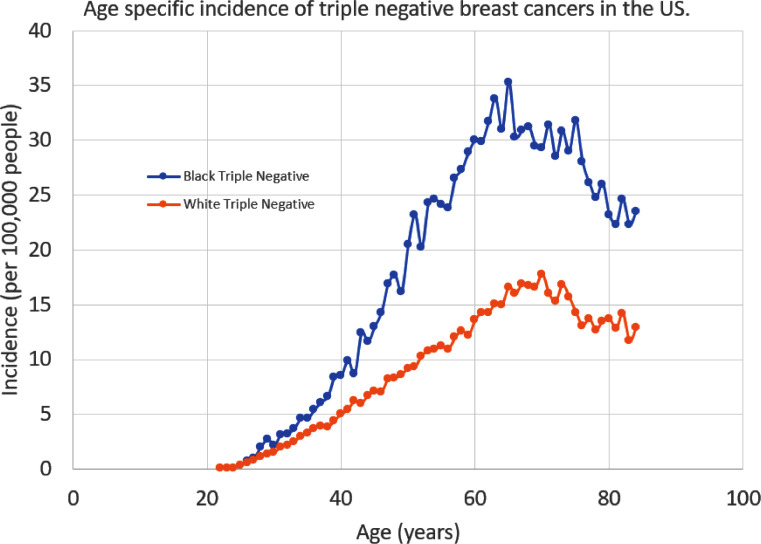
The age specific incidence for triple negative breast cancer in two race categories: white and Black people. This data is from SEER 21 cancer registries over the period of 2000 to 2016.

The manuscript by Saleh et al. [1] takes a first step in that direction. The authors provide a molecular characterization of 15 Bantu patients from Kenya, all of whom were diagnosed with triple negative breast cancer. The authors compare this molecular characterization to similar characterizations from 19 Black and 23 white women from the US, all of whom have been diagnosed with similar triple negative breast cancers. From this comparison, they identify genetic signatures that are specific to the Kenya cases. These signature genes undergo significant differential regulation in the Kenya cases, but show no differential regulation in the Black and white breast tumor tissue nor in normal breast tissue.

This study begins to reveal that breast cancer incidence and outcome varies depending on the genetics of the patient. This phenomenon has mostly been studied in white Americans, with some work done characterizing Black Americans. However, little work has been done on the many, many, different people of Africa. Studies like this one could add significantly to our understanding of cancers.

## Author's contribution statement

James P Brody is the sole author of this work. He conceptualized the project, analyzed the SEER data, and wrote and edited both the initial and final draft of the manuscript.

## Declaration of Competing Interest

The authors declare that they have no known competing financial interests or personal relationships that could have appeared to influence the work reported in this paper.
